# Photoperiods induced the circRNA differential expression in the thyroid gland of OVX+E_2_ ewes

**DOI:** 10.3389/fendo.2022.974518

**Published:** 2022-08-29

**Authors:** Wei Wang, Xiaoyun He, Ran Di, Xiangyu Wang, Mingxing Chu

**Affiliations:** Key Laboratory of Animal Genetics, Breeding and Reproduction of Ministry of Agriculture and Rural Affairs, Institute of Animal Science, Chinese Academy of Agricultural Sciences, Beijing, China

**Keywords:** sheep, thyroid gland, photoperiod, circRNA, miRNA

## Abstract

Circular RNAs (circRNAs) are non-coding RNAs newly identified and play important roles in RNA regulation. However, little is known regarding photoperiods induced circRNAs in the thyroid gland. In this study, we performed a comprehensive analysis of circRNA profiles in the thyroid gland of OVX+E_2_ ewes at different photoperiods by whole transcriptome sequencing. A total of 37,470 novel circRNAs were detected in different photoperiods (42 days of short photoperiod treatment, SP42; 42 days of long photoperiod treatment, LP42; SP42 transfer to LP42, SPLP42), with a total of 817 circRNAs for SP42-LP42 (down: 132; up: 114), LP42-SPLP42 (down: 136; up: 112) and SP42-SPLP42 (down: 182; up: 141) having differentially expressed. Functional enrichment annotation analysis of DE-circRNAs for GO and KEGG by R package, features that influence photoperiod response in Sunite ewes through the Inositol phosphate metabolism, cGMP-PKG signaling pathway, Calcium signaling pathway, MAPK signaling pathway, and Oocyte meiosis. In addition, competitive endogenous RNA (ceRNA) network analysis revealed target binding sites for identified miRNAs in DE-cirRNAs such as oar-miR-10b, oar-miR-200c, oar-miR-21, oar-miR-370-3p, oar-miR-377-3p, oar-miR-181a, oar-miR-432, and oar-miR-495-3p. These results of this study will provide some new information for understanding circRNA function as well as the changes in the sheep thyroid gland under different photoperiods.

## Introduction

Photoperiod induction is a key external factor for organisms to reproduce seasonally under different light levels throughout the year to maximize the survival of their offspring. Most ewes in China are seasonally estrus with normal ovulatory cycles, which is controlled by photoperiod (the length of the daily light phase) (1). The light exposure is one of periodic changes in the external environmental factor, and many endocrine factors are known to show environmental-related changes. TSH (thyroid-stimulating hormone) secretion, as well as the hypothalamic-pituitary-thyroid (HPT) axis, exhibit a pronounced diurnal rhythm and regulated circadianly by the suprachiasmatic nucleus (SCN) of the anterior hypothalamus (2). Under short photoperiod, melatonin inhibits the pars tuberalis production of TSHβ, which in turn acts on tanycytes to regulate the deiodinase 2/3 balance resulting in a finely tuned seasonal control of the intrahypothalamic thyroid hormone triiodothyronine (T3) (3). T3 and T4 (thyroxine) are produced by differentiated cells within the thyroid follicles, known as thyrocytes (4). The thyroid gland plays a crucial role in organismal development and homeostasis (5). In mammals, it is late in organogenesis that progenitors cells differentiate into follicular cells and begin to produce hormone. Prior to this, thyroid-dependent embryonic and fetal development of the organism rely entirely on maternal T4 supply (6). Recent evidence suggests that the thyroid gland plays an important role in endogenously generated reproductive conversion in certain animals that present seasonal breeding (7). Thyroid hormone (TH) directs seasonal breeding through reciprocal regulation of TH deiodinase (*Dio2*/*Dio3*) gene expression in tanycytes in the ependymal zone of the medio-basal hypothalamus (MBH) (8). In sheep, thyroidectomy (THX) had no clear effect on the transition to late summer reproduction while blocked the late winter transition to non-breeding (9). Therefore, understanding how the thyroid gland, the only source of thyroid hormones in the body (4), changes under different photoperiods is critical to understanding seasonal reproduction in mammals.

Circular RNAs (circRNAs) are stable endogenous biomolecules that is not susceptible to degradation by RNase and have a covalently closed structure due to the absence of 5′ end caps or 3′ poly(A) tails (10, 11). As sequencing methods and technologies have advanced and improved, researchers have found that circRNAs are highly abundant in eukaryotes and many of them are evolutionarily conserved. In metazoans, circRNAs are expressed in a tissue-specific manner and are highly stable and able to accumulate with age in neural tissue (12–14). Related studies have shown that certain circRNAs interact with microRNAs, and in addition circRNAs can regulate immune responses and behavior (15). There is a growing interest in the functions circRNAs play in animals, and it is now known that circRNAs have a wide range of biological functions from regulation of gene expression to protein-coding and mRNA competition (16). CircRNAs have a more stable closed-loop covalent structure than other RNAs, making them suitable for future development as biomarkers (17). This is particularly important. Furthermore, there is growing evidence that circRNAs are useful molecules that can provide therapeutic targets for a variety of diseases (18, 19), and that circRNAs play a role in the development of germ cells (20, 21). To date, the analysis of circRNAs and their role in thyroid tissue under different photoperiods remains completely unknown.

Currently, there is a gap in scientists’ understanding of the molecular mechanisms of photoperiodic regulation of the molecular neuroendocrine axis and seasonal changes in reproduction. Even though some studies have identified expression patterns for key genes or proteins under different light conditions (22, 23), a comprehensive understanding of the role played by circRNAs in the thyroid gland under different photoperiods is lacking. The OVX models have been widely used and progressed in previous mammalian studies, such as rats, mice, goats, and sheep (24, 25). The present study builds on our work to analyze the effects of photoperiodic changes on the thyroid gland transcriptome through bioinformatics approaches (23). The findings of this study will provide new information for understanding circRNA function as well as the changes in the sheep thyroid gland under different photoperiods.

## Material and methods

### Sample collection

All samples were obtained from a group of nine Sunite ewes (clinically normal and non-pregnant) with OVX+E_2_ treatment (25–27). Briefly, the estradiol treatment had an inner diameter of 3.35 mm and an outer diameter of 4.65 mm and was filled with 20 mg of crystalline 17β estradiol (Sigma Chemical Company, St. Louis, MO). The implants were inserted into the axillary region and designed so that E2 circulated at levels of approximately 3-5 pg/ml for 2 weeks. Sheep aged 2-3 years were randomly assigned to one of the three rooms representing different treatments: (1): Short Photoperiod, SP, 8/16 h light-dark; (2): Long Photoperiod, LP, 16/8 h light-dark; (3): Short Photoperiod transfer to Long Photoperiod, SP-LP. The nine ewes were reared using standard techniques (same feeding management and living environment, fed ad libitum, and free access to water) on a farm in the Institute of Animal Husbandry and Veterinary Medicine, Tianjin Academy of Agricultural Sciences, Tianjin, China. The thyroid glands were collected from a total of ewes in three rooms at SP42 days, LP42 days, and SP-LP42 days (42 days of short photoperiod treatment transfer to 42 days of long photoperiod treatment) after the slaughter, frozen in liquid nitrogen, and stored at -80°C for subsequent transcriptome sequencing.

### RNA extraction and sequencing

Total RNA was isolated from samples using the TRIzol reagent (Invitrogen, Carlsbad, CA, USA) according to the manufacturer’s instruction. RNA purity was confirmed with the NanoPhotometer spectrophotometer (IMPLEN, CA, USA), and concentration and integrity were assessed using electrophoresis and the RNA Nano 6000 Assay Kit of the Bioanalyzer 2100 system (Agilent Technologies, Santa Clara, CA, USA). The RIN value of 7.5 or higher indicates that the sample is satisfactory and can proceed to the next step of analysis. Subsequently, ribosomal RNA (rRNA) was then removed from the total RNA to enrich circRNAs, and sequencing libraries were prepared for the nine rRNA-removed samples using NEBNext^®^ UltraTMDirectional RNA Library Prep Kit for Illumina^®^ (NEB, Ipswich, MA, USA) for rRNA-depleted RNA. Finally, these libraries were sequenced on the Illumina platform.

### Bioinformatics analysis

Reads adapter sequences, ploy-N, and low-quality reads were removed to obtain clean data. Simultaneously, the Q20 and Q30 of the clean data were calculated. The reference genomes and the annotation file were downloaded from the ENSEMBL database (http://www.ensembl.org/index.html). Reads were mapped to reference genome by the BWA-MEM method (28) using bwa (0.7.9a), which allows fast and efficient to align reads and mapping of fragment reads to genomes.

### Identification of circRNA

CIRI (29) is an efficient and fast circRNA identification tool. It first uses the BWA-MEM algorithm to perform sequence splitting and comparison, then analyzes the resulting SAM files for PCC (paired chiastic clipping) and PEM (paired-end mapping) sites, as well as the GT-AG splicing signal. And GT-AG clipping signals, and finally the sequences with splicing sites were re-matched using a dynamic programming algorithm to ensure the identification of circRNAs. The sequences with splicing sites were then re-matched using a dynamic programming algorithm to ensure the reliability of the identified circRNAs.

### Differential expression analysis

The amount of circRNA expression is measured by the number of nodal reads referring to SRPBM (Spliced Reads per Billion Mapping). Where SR is the number of spliced Reads, N is the total number of mapped reads in a given sample.


$$SRPBM=\frac{SR*10^{9}}{N}$$

DEGseq (http://www.bioconductor.org/packages/release/bioc/html/DEGseq.html) and DESeq (http://www.bioconductor.org/packages/release/bioc/html/DESeq.html) were used for differential expression analysis of two samples with or without replicates. Under the assumption that the number of reads coming from a gene (or transcript isoform) follows a binomial distribution, DEGseq is proposed based on the MA-plot and widely used for differential expression analysis. The P-value could be assigned to each gene and adjusted by BH. Genes with q ≤ 0.05 and |log2_ratio|≥1 are identified as differentially expressed genes.

### Functional enrichment analysis

Functional enrichment analysis was implemented by GOseq R package (30). The potential functions of the parental genes of differential circRNAs were analyzed by GO (Gene Ontology, http://geneontology.org/) and KEGG (Kyoto Encyclopedia of Genes and Genomes, http://www.kegg.jp/) pathway functional annotation. GO terms with q<0.05 are considered to be significantly enriched. In the same method with GO enrichment analysis, significantly enriched KEGG pathways are identified.

### Construction of integral circRNA-mRNA interaction networks

To predict the function of DE circRNAs and their target miRNAs in sheep reproduction, a network based on circRNAs and miRNAs was constructed using Cytoscape (31) (V3.8.2).

### Real-time PCR

To confirm the sequencing results, the expression of six circRNAs was verified by real-time PCR. After extraction of total RNA, samples were reverse transcribed to produce cDNA, cDNA synthesis was performed according to the supplier’s instructions. Two microlitres of each cDNA were amplified by PCR using specific primers. The PCR efficiency of each RNA was estimated by calculating a standard curve using a serial dilution of four cDNA spots. The cycle threshold (Ct) was converted to a quantity using the comparative Ct method, setting the relative amount of three groups for each gene to 1 (quantity = 10^-ΔCt^/slope). Data normalization was performed using the Actin reference gene. The correlation between sequencing and PCR results was calculated using correlation tests.

## Results

### Summary statistics of RNA-seq data in thyroid gland

**Figure 1:**
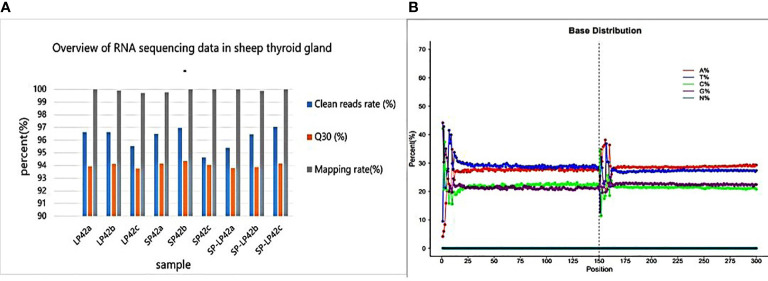
Overview of RNA sequencing data in thyroid glands. **(A)** Clean read rate, Q30 and mapping rate in sheep thyroid gland. **(B)** Base distribution of circRNAs.

As shown in **Figure 1**, each sample of RNA sequencing of nine sheep thyroid glands produces raw reads of more than 11.36G, with at least 11.06 (95.54%), 11.53 (94.64%), and 10.83 (95.39%) million clean reads for LP42T, SP42T, and SP-LP42T, respectively (**Supplementary Table 1**). After removing low-quality sequences, the Q30 scores of the sample data were not less than 93.76%. In addition, 99.70%-99.99% was mapped to the sheep reference genome (*Oar_v4.0*). Interestingly, the G + C contents of the circRNAs identified in different photoperiods were around 40%. These showed that the RNA sequencing data were highly credible.

### Characterization of circRNAs

**Figure 2:**
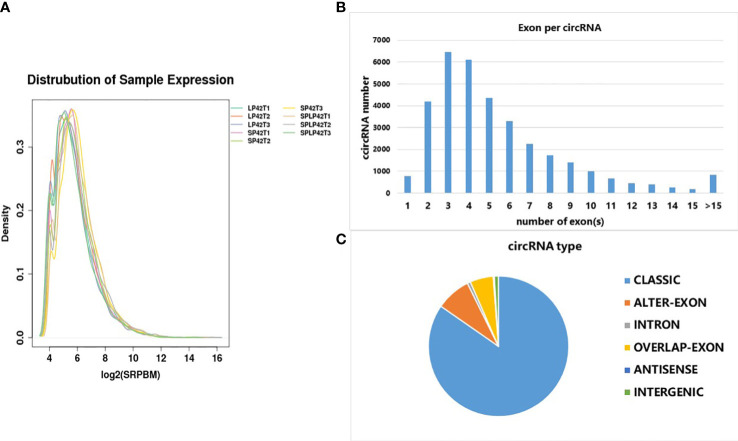
Characterization of circRNAs identified at different photoperiods of ovine thyroid gland. **(A)** FPKM distribution of each sample. **(B)** The number of exons in each circRNA. **(C)** The number of different types of circRNA.

The results revealed similar patterns of expression distribution in nine samples. A total of 37470 novel circRNAs were detected in different photoperiods were analyzed (**Supplementary Data 1** and **2**). The distribution trend of expression in nine samples was similar (**Figure 2A**). The circRNAs were identified with 6452 containing three exons, 6094 containing four exons, 4354 containing five exons and 4200 containing two exons (**Figure 2B**). The circRNAs were divided into six classes, classic, alter-exon, intron, overlapped-exon, antisense, and intergenic; among these, intergenic circRNAs were the most common (85%, **Figure 2C**).

### Differentially expressed cirRNAs of three photoperiods in the ovine thyroid gland

**Figure 3:**
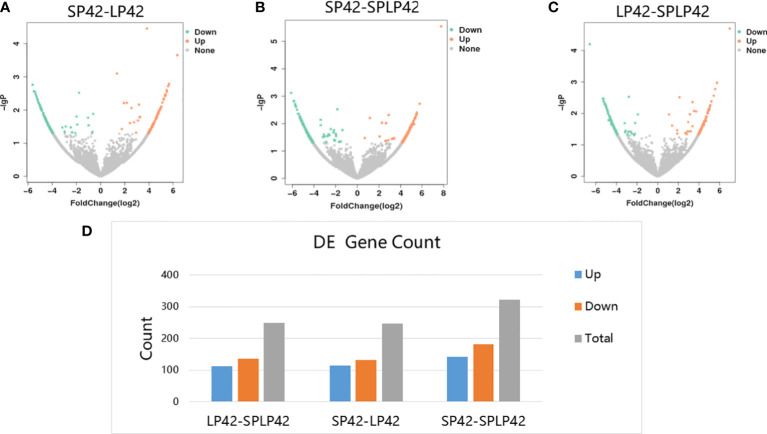
Analysis of circRNAs differentially expressed in the different photoperiods of ovine thyroid gland. **(A)** Volcano map of differentially expressed circRNA in SP42-LP42. **(B)** Volcano map of differentially expressed circRNA in SP42 and SPLP42. **(C)** Volcano map of differentially expressed circRNA in LP42-SPLP42. **(D)** Analysis of circRNAs differentially expressed in LP42-SPLP42, SP42-LP42 and SP42-SPLP42.

A total of 817 circRNAs were differentially expressed in LP42-SPLP42, SP42-LP42 and SP42-SPLP42. The expressions of 248 circRNAs (down: 136; up: 112) were different in LP42-SPLP42; the expressions of 246 circRNAs (down: 132; up: 114) were different in SP42-LP42; the expressions of 323 circRNAs (down: 182; up: 141) were different in SP42-SPLP42 (**Figure 3**, **Supplementary Data 3**).

### CircRNA source gene analysis and differentially expressed circRNAs analysis

**Figure 4:**
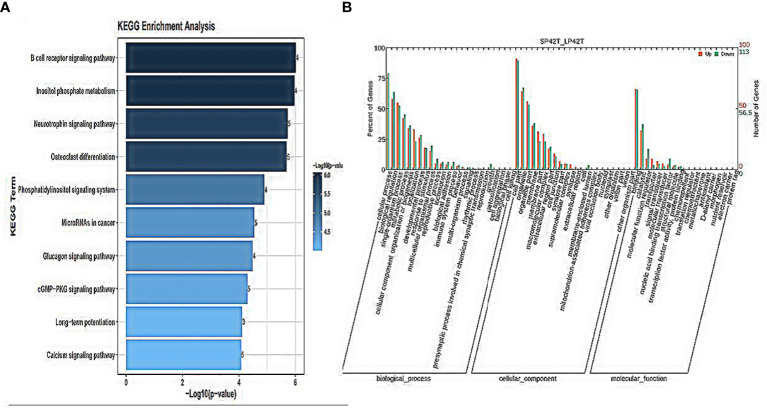
An analysis of GO and KEGG enrichment in the thyroid gland was performed. **(A)** Ten major KEGG enrichment pathways of DE-circRNAs were observed in SP42-LP42. **(B)** Analysis of GO function of DE-circRNAs in SP42-LP42.

**Figure 5:**
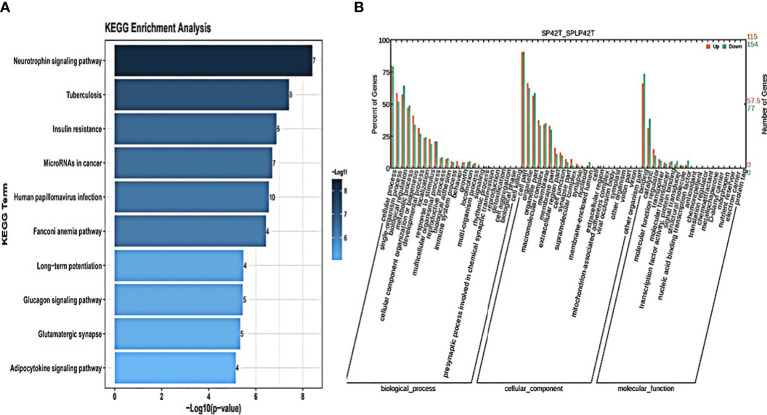
An analysis of GO and KEGG enrichment in the thyroid gland was performed. **(A)** Ten major KEGG enrichment pathways of DE-circRNAs were observed in SP42-SPLP42. **(B)** Analysis of GO function of DE-circRNAs in SP42-SPLP42.

**Figure 6:**
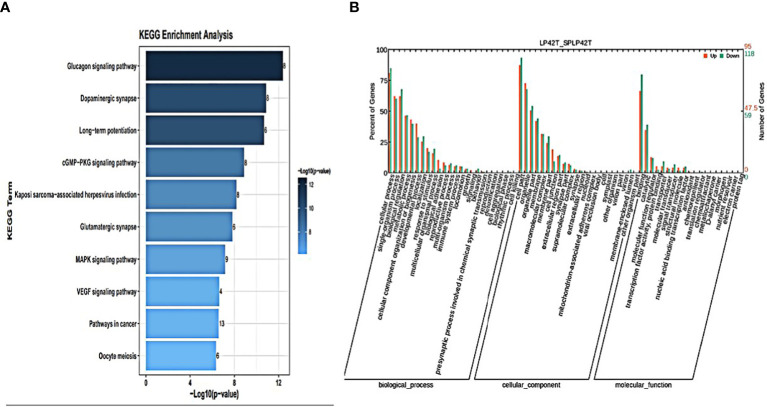
An analysis of GO and KEGG enrichment in the thyroid gland was performed. **(A)** Ten major KEGG enrichment pathways of DE circRNAs were observed in LP42-SPLP42. **(B)** Analysis of GO function of DE-circRNAs in SP42-SPLP42.

GO annotation and KEGG pathways annotation analysis was performed on the source genes of the differentially expressed circRNAs to identify the top ten GO terms and KEGG enrichment pathways with a high level of confidence. Further, differentially expressed circRNAs enrichment (*p*< 0.05) was seen in KEGG pathways. KEGG analysis showed that the most enriched pathways specific for differentially expressed circRNAs in SP42 and LP42 were B cell receptor signaling pathway, the Inositol phosphate metabolism, Neurotrophin signaling pathway, and Osteoclast differentiation (**Figure 4A**). Between SP42 and SPLP42, the most significantly enriched pathway is Neurotrophin signaling pathway and Tuberculosis also enriched. Several pathways are noteworthy, such as MicroRNAs in cancer, Insulin resistance, Glucagon signaling pathway, and Glutamater synapse (**Figure 5A**). In LP42 and SPLP42, the most interesting pathways were those associated with reproduction (Oocyte meiosis, MAPK signaling pathway, and Dopaminergic synapase, as shown in **Figure 6A**), and pathways associated with the disease were also enriched (cGMP-PKG signaling pathway, Kaposi sarcoma-associated herpesvirus infection, VEGF signaling pathway and Pathways in cancer). In addition, comparative GO analysis was performed to find specific functional terms over-represented in up- and down-regulated circRNAs, a. The top ten significantly enriched GO terms belonged to the three GO types for DE circRNAs: imolecular function (MF), cellular component (CC), and biological process (BP),. DE circRNAs enriched source genes with terms related to the cell part, binding, and the cellular processes (**Figures 4B**, **5B**, **6B**).

### CircRNA-miRNA co-expression network construction

Based on the prediction to interact with correlation circRNA-miRNAs, a circRNA-miRNA co-expression network was constructed to explore the molecular mechanisms by which different photoperiods affect the transcriptome of the sheep thyroid glands.

**Figure 7:**
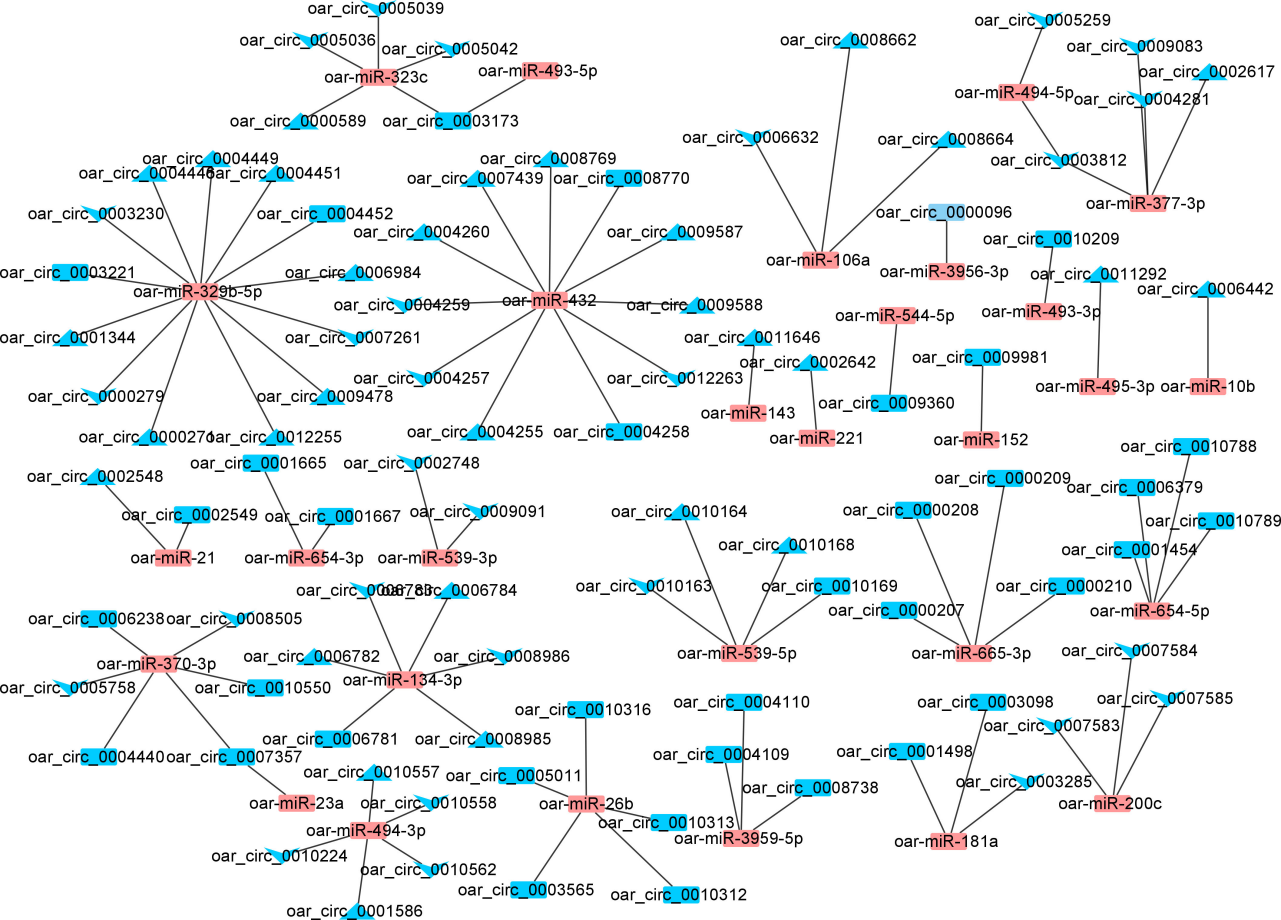
The networks of circRNA-miRNA for SP42-LP42. Blue and orange represent miRNAs and circRNAs, respectively. Triangle and V represent upregulated and downregulated, respectively.

In the SP42 and LP42 groups, a total of 36 DE miRNAs and 98 DE circRNAs (59 up-regulated and 65 down-regulated circRNAs) were involved in the network (**Figure 7**). Notably, oar_circ_0007357 was also found to target both oar-miR-23a and oar-miR-370-3p, as both genes are important for seasonal reproduction. Moreover, oar-miR-370-3p was also more abundant in the network and was expected to interact with 10 circRNAs (up-regulated). In the SP42 and SPLP42 groups, a total of 29 DE miRNAs and 105 DE circRNAs (57 up-regulated and 79 down-regulated circRNAs) were involved in the network (**Supplementary Figure 1**). Notably, oar_circ_0003811 and oar_circ_0003812 were also found to target both oar-miR-377-3p and oar-miR-494-5p. Moreover, oar-miR-377-3p was also more abundant in the network. The network of LP42 and SPLP42 contained 32 differentially expressed lncRNAs and 112 target genes, with proposed a trans relationship between oar-miR-133 and oar_circ_0007178 (**Supplementary Figure 2**). Oar-miR-329b-5p and oar-miR-432 had the most circRNA binding sites (**Figure 7**, **Supplementary Figures 1**and **2**, **Supplementary Data 4**).

### Quantitative RT-PCR validation

**Figure 8:**
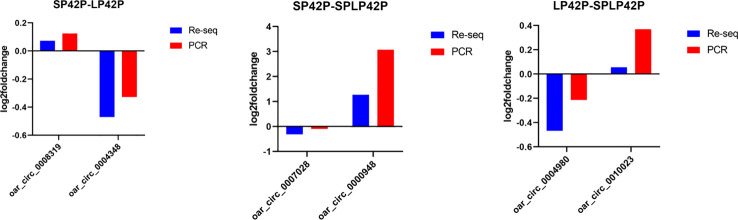
Validation of RNA-Sequencing (RNA-seq) data using PCR. RNA-Seq and PCR results of three selected expressed circRNAs in the thyroid gland of Sunite sheep at different photoperiods.

To validate the sequencing results, the same RNA samples used for sequencing were tested for expression of 6 circRNAs (oar_circ_0008319, oar_circ_0004348, oar_circ_0007028, oar_circ_0000948, oar_circ_0004980 and oar_circ_0010023) using real time PCR (**Supplementary Data 5**). The expression profiles of these RNAs detected by real time PCR were similar to those obtained by sequencing (**Figure 8**), which confirmed the reliability of the sequencing results.

## Discussion

Thyroid hormones regulate the metabolism and development of the ovarian, uterus and placental tissues and therefore play a vital role in the proper functioning of the female reproductive system (32). Previous studies have shown that TH signaling is an important component of the hypothalamic mechanisms that drive the seasonal reproductive cycle (8). Specifically, it is during the specific breeding seasons that the HPG axis is activated in seasonally breeding animals (33). As discussed previously, THX has been found to prevent the response of the reproductive systems of some species to the duration of light (34–36). It is clear that the activated state of thyroid hormone metabolism is central to the regulation of photoperiodic responses and that the role of thyroid tissue is clearly essential (37). And genes involved in photoperiodic signaling pathways could be useful targets for domesticating wildlife (38). Some evidence already suggested that circRNAs play vital roles in epigenetic regulation and embryonic development (39, 40). Therefore, in this study, we performed transcriptome analysis of thyroid tissues under different photoperiods in order to be able to screen for new photoperiod-induced candidate potentially competitive endogenous RNAs (ceRNAs).

CircRNAs are known to promote the transcription of source genes by interacting with U1 small ribonucleoproteins (snRNPs) and RNA polymerase II (41). Therefore, identification of the potential functions of source genes associated with circRNAs may shed light out on their functions (42). In this study, the source genes of differentially expressed circRNAs found to been riched in Inositol phosphate metabolism, cGMP-PKG signaling pathway, Calcium signaling pathway, MAPK signaling pathway and Oocyte meiosis. Inositol phosphate is known to be an essential nutrient for cell growth, survival and embryonic development, and inositol metabolism is associated with embryonic development (43). Studies have shown that Cryptobacterium elegans IPMK-1 (homologous to mammalian inositol polyphosphate multi-kinase) regulates rhythmic behavior in animals, and inositol polyphosphate multi-kinase IPMK-1 regulates development by modulating calcium signaling pathway in Cryptobacterium hidradenum (44). Calcium signaling pathways are an important KEGG term associated with prolificacy (45). The cGMP-PKG signaling pathway and MAPK signaling pathway were significantly enriched in the LP42-SPLP42 group, which also validates the specific expression of the gene in summer biology compared to winter biology (24).

In addition, circRNAs can also directly regulate protein synthesis *via* mRNA, which has been found to have potential effects on ovarian follicles in ewes (46). *CAMK4*, *PPP3CC*, *TSHR*, *PDE5A*, *ITPR1*, *PPP3CB*, *PLCB1*, *DUSP16*, and other circRNA source genes enriched in the above reproduction-related pathways were significantly upregulated. *CAMK4* is a candidate domestication gene locus, associated with a wide range of traits (morphology, immunity, reproductive or production traits) in sheep (47). It has long been shown that thyroid-stimulating hormone receptor (TSHR) is associated with reproduction and plays an important role in metabolic regulation and photoperiodic control during reproduction in birds and mammals (48, 49). Furthermore, 29 bp nucleotide sequence variation within the *TSHR* was significantly associated with litter size in sheep. In our experiments, the *TSHR* was significantly up-regulated in SP-LP, suggesting a role in the photoperiod, in agreement with previous findings (50). It is noteworthy that *PPP3CC* was different and significantly up-regulated in three periods, the expression of *PPP3CC* in the testis is critical for sperm viability and male fertility (51). In addition, *PPP3CC* and *ITPR1* have also been associated with reproduction in cattle (52, 53), but so far no studies have been found on *PPP3CC* in seasonal estrus in sheep. Elevated levels of *PPP3CB* in MII oocyte CC may be a useful indicator of the fertilizing potential of selected oocytes (53, 54). Using zebrafish as a model, *PDE5A* was found to be expressed only in oocytes and was able to maintain oocyte maturation (55). *PLCB1* was identified as a candidate gene for litter size (56). Significant changes in the expression of *DUSP16* during the ovulation cascade may regulate its course (57). The above enrichment pathways, and their associated genes, are therefore important for seasonal reproduction. Our study shows that persistently altered circRNAs are involved in cGMP-PKG, MAPK, Oocyte meiosis and Calcium signaling pathways, suggesting that circRNAs may act through these pathways in thyroid glang development and seasonal estrus in sheep.

In recent years, different types of ncRNAs in organisms, such as miRNA (58), lincRNA (59) and circRNA (60) have been identified and shown to play critical roles. The function of circRNA is usually studied together with their miRNA sponges (61). In this study, we constructed a circRNA-miRNA network map of the differences between the three comparison groups and found multiple miRNAs recurring in it, such as oar-miR-10b, oar-miR-200c, oar-miR-21, oar-miR-370-3p, oar-miR-377-3p, oar-miR-181a, oar-miR-432 and oar-miR-495-3p. Among them, oar_circ_0007583, oar_circ_0007584 and oar_circ_0007585 are competitive endogenous RNAs of oar-miR-200c, can affect the TGF-β signaling pathway to increase the efficiency of somatic cell reprogramming in sheep (62). In male species with seasonal reproduction, TGF-β signaling, a molecular pathway controlling the adhesion function of spermatogenic epithelial cells, is altered during the regression process (63). This also shows that our study is consistent with the above results that differentially expressed miRNAs function under different photoperiods, with circRNAs acting as miRNA sponges to play a regulatory role. Oar_circ_0002617, oar_circ_0003812, oar_circ_0004281 and oar_circ_0009083 are competitive endogenous RNAs of oar-miR-377-3p. Oar-miR-377-3p is related to oocyte quality, this can directly affect the ability of the embryo to develop (64). Oar-miR-181a, oar-miR-432 and oar-miR-495-3p were identified to be associated with reproductive function (42, 65). Oar-miR-21, oar-miR-370-3p, and oar-miR-10b are differentially expressed in sheep-related diseases (66, 67), which have not been studied in seasonal reproduction, their differential expression under different photoperiods deserves further study. In summary, oar_circ_0007583, oar_circ_0007584 and oar_circ_0007585 are potentially competitive endogenous RNAs (ceRNAs) that can regulate gene transcription and can function under different photoperiods in sheep.

## Conclusion

In this study, circRNA expression profiles were established for the thyroid gland of Sunite ewes under different photoperiods. Key circRNAs involved in photoperiod response were identified to regulate sheep thyroid development through signaling pathways such as Inositol phosphate metabolism, cGMP-PKG signaling pathway, Calcium signaling pathway, MAPK signaling pathway, and Oocyte meiosis. Meanwhile, analysis of competitive endogenous RNA networks revealed miRNA targets of oar-miR-10b, oar-miR-200c, oar-miR-21, oar-miR-370-3p, oar-miR-377-3p, oar-miR-181a, oar-miR-432 and oar-miR-495-3p among circRNAs. It appears that circRNAs mainly act as sponges for several reproduction-related miRNAs, which in turn influence seasonal reproduction in sheep.

## Data availability statement

The data presented in the study are deposited in the NCBI repository under the accession number PRJNA856274 (https://www.ncbi.nlm.nih.gov/sra/PRJNA856274).

## Ethics statement

The animal study was reviewed and approved by Science Research Department of the Institute of Animal Sciences, Chinese Academy of Agricultural Sciences.

## Author contributions

WW and XH performed the experiments, analyzed data, and wrote the first draft. RD and XW provided analysis tools and Data interpretation, MC contributed to the experimental design and manuscript revision. All authors contributed to the article and approved the submitted version.

## Funding

This research was funded by the National Natural Science Foundation of China (32172704, and 32102508), the Earmarked Fund for China Agriculture Research System of MOF and MARA (CARS-38), the Central Public-Interest Scientific Institution Basal Research Fund (No.2021-YWF-ZYSQ-14), the Open Project of Jiangsu Key Laboratory of Animal genetic Breeding and Molecular Design (AGBMD2020), the Agricultural Science and Technology Innovation Program of China (CAAS-ZDRW202106 and ASTIP-IAS13).

## Conflict of interest

The authors declare that the research was conducted in the absence of any commercial or financial relationships that could be construed as a potential conflict of interest.

## Publisher’s note

All claims expressed in this article are solely those of the authors and do not necessarily represent those of their affiliated organizations, or those of the publisher, the editors and the reviewers. Any product that may be evaluated in this article, or claim that may be made by its manufacturer, is not guaranteed or endorsed by the publisher.
