# Supplementary material for: Photoperiods induced the circRNA differential expression in the thyroid gland of OVX+E2 ewes
Source: Front Endocrinol (Lausanne). 2022 Aug 29;13:974518. doi: 10.3389/fendo.2022.974518 (PMC9464909; doi:10.3389/fendo.2022.974518)
Supplement: Supplementary file 1 [file DataSheet_1.docx]

Supplementary Material

# Supplementary Figures and Tables

## Supplementary Figures


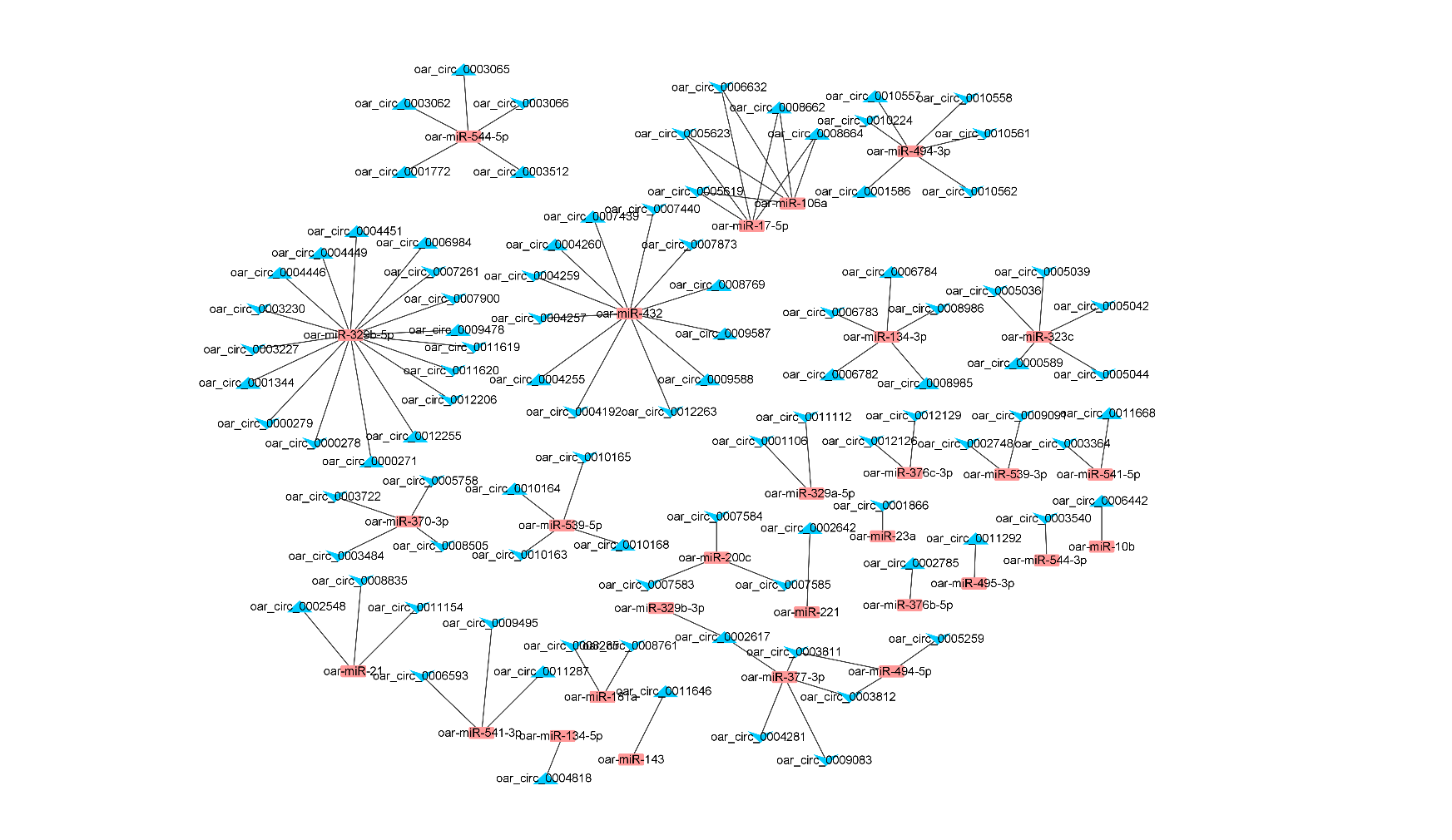


**Supplementary Figure 1.** The networks of circRNA-miRNA for SP42-SPLP42. Blue and orange represent miRNAs and circRNAs, respectively. Triangle and V represent upregulated and downregulated, respectively.


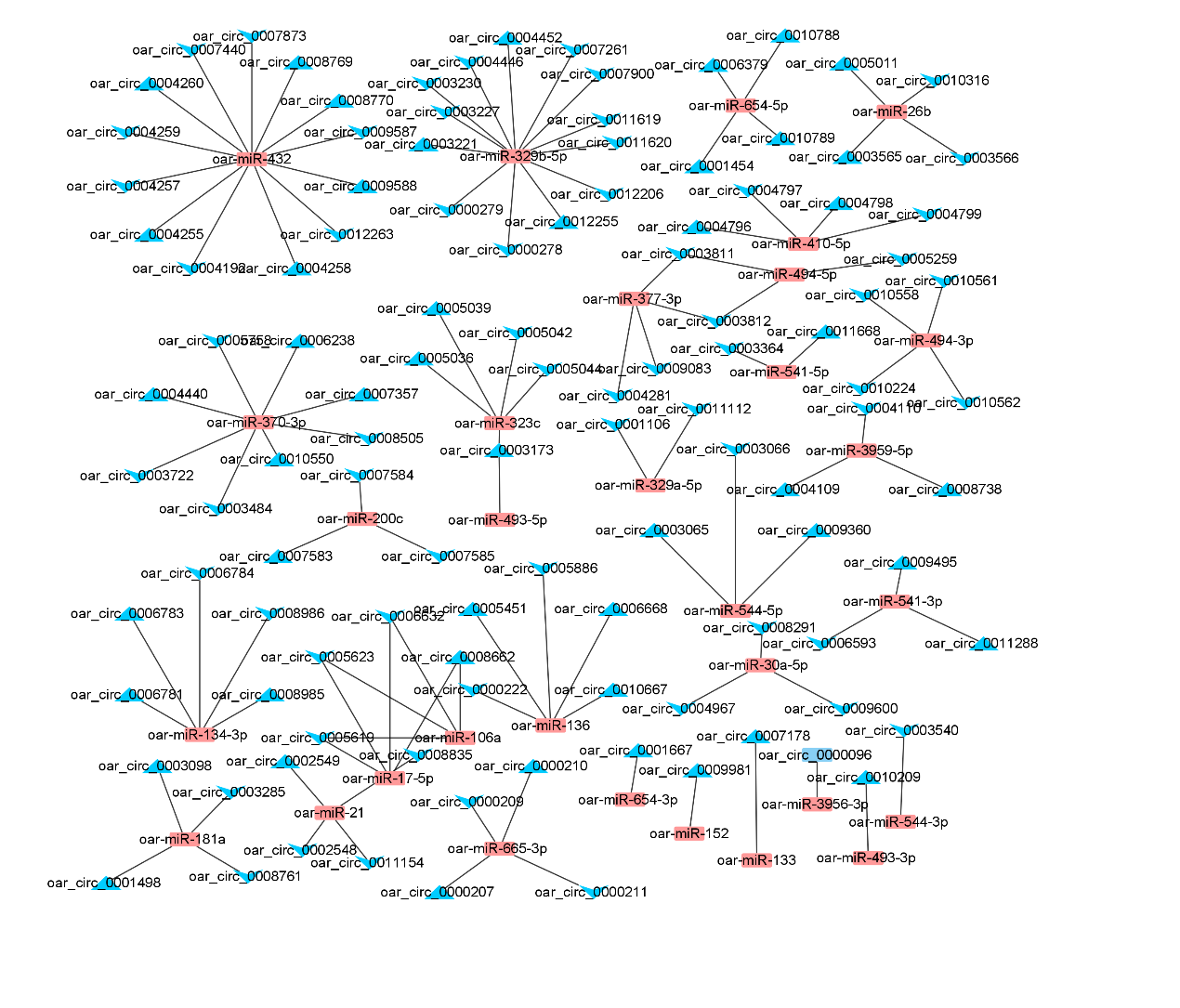


**Supplementary Figure 2.** The networks of circRNA-miRNA for LP42-SPLP42. Blue and orange represent miRNAs and circRNAs, respectively. Triangle and V represent upregulated and downregulated, respectively.

## Supplementary Tables

| **Table 1 Overview of RNA sequencing data of three photoperiods in sheep thyroid gland** | | | | | | |  |
| --- | --- | --- | --- | --- | --- | --- | --- |
| Sample name | Raw reads | Clean reads | Clean reads rate (%) | Q30 (%) | Mapping rate(%) | circRNA number |  |
|  |  |  |  |  |  |  |  |
| LP42T1 | 125,086,648 | 120862882 | 96.62 | 93.95 | 99.98 | 4774 |  |
| LP42T2 | 114,464,214 | 110619664 | 96.64 | 94.15 | 99.9 | 4124 |  |
| LP42T3 | 127,822,204 | 122122532 | 95.54 | 93.76 | 99.7 | 3650 |  |
| SP42T1 | 128,205,226 | 123710152 | 96.49 | 94.17 | 99.76 | 3853 |  |
| SP42T2 | 124,015,570 | 120265994 | 96.98 | 94.35 | 99.98 | 3238 |  |
| SP42T3 | 121,918,860 | 115388502 | 94.64 | 94.05 | 99.98 | 4046 |  |
| SP-LP42T1 | 113,613,174 | 108371314 | 95.39 | 93.79 | 99.98 | 3542 |  |
| SP-LP42T2 | 132,383,336 | 127709202 | 96.47 | 93.88 | 99.87 | 5416 |  |
| SP-LP42T3 | 132,355,318 | 128440756 | 97.04 | 94.17 | 99.99 | 4827 |  |
| LP42T1 represents thyroid gland (T) sample “1” in SP42. | | | | | | |  |
